# The Earthquakes in Calabria

**Published:** 1905-09-23

**Authors:** 


### The Earthquakes in Calabria.

The appalling catastrophe which has brought ruin and destruction upon the two southern provinces of Calabria, a catastrophe only comparable to the great earthquake at Casamicciola in 1883, or to the destruction of St. Pierre by the eruption from Mont Pelée, is one calculated to excite the most lively sympathy among all civilised nations, and to call upon them for such timely help as may serve in some degree to mitigate the immediate effects of the calamity. The population of the two provinces chiefly affected cannot be much less than a million; and, with the exception of a comparatively small mining industry, it is chiefly agricultural, and agricultural after a very primitive fashion, such as would naturally be engendered by the fertility of the soil and the geniality of the climate. Over an area of some five thousand square miles the shocks seem to have been almost universal, and from every side we hear of buildings levelled to the ground, of deaths the full tale of which has still to be counted, of vast numbers of maimed and otherwise injured persons, of thousands left without shelter upon the hillsides, and of a general destruction both of food supplies and of the organisations by which they could be furnished. The principal towns of both provinces, Catanzaro, Monteleone, Reggio, Palmi, have all suffered more or less severely, and are all more or less disabled from rendering assistance to the dwellers in the rural districts and in the mountains. There must be immediate want of everything—of food, of tents, of medicines, of surgeons—not to speak of the prospective want of building materials and of seed, for the restoration of the dwellings and of the crops. On many former occasions of great national calamity the consequent distresses have called forth in this country not only the practical sorrow of the Quaker, the being "sorry five shillings," but also the necessary supply of heads and hands by which alone the mere money donations could be rendered effective. Italy has for many years been the firm and consistent friend of England, and there are many conceivable conditions in which her friendship might be of the greatest possible value. We trust that the opportunity of cementing it which is afforded by her present misfortunes will not be thrown away, that our national contributions to the relief fund will be commensurate with the urgency of the occasion, and that they will be supplemented by those personal efforts which are alike more useful and more highly appreciated than any gifts of money alone. Few things are more remarkable than the scantiness of the details which so far have been received from the stricken localities. Either the panic produced by the earthquakes, in themselves the most utterly demoralising and paralysing of all the risks to which humanity can be exposed, has tied the tongues and pens of those from whom descriptions might have been expected, or the urgency of the immediate claims upon everybody in the localities affected has left them no time in which to supply narratives of the sufferings around them. The latter is, we fear, the more likely solution of the question, and, if so, it is one that should appeal to us even more strongly than any number of letters or of descriptive articles. The reported loss of life leads to the belief that the Calabrians, although dwellers in a region in which earthquakes are common, have hitherto escaped visitations of great severity, and that the construction of their houses has not been governed by any estimate of the probable consequences of their overthrow. In Japan, as we know, the frequency of comparatively trifling earthquakes has led to the general adoption of one-storied buildings of the flimsiest construction, and, where greater stability has been required, as for example, in the National Bank at Tokio, this has been gained by the construction of a massive platform upon which the building has been placed, and to which earth vibration is not readily imparted by the alluvial soil around. In Calabria, as at Lisbon, a serious earthquake may be an isolated phenomenon, and human nature being what it is, we may be certain that this expectation will be entertained and largely acted upon. It need not, nevertheless, prevent the work of reconstruction from being based upon principles the observance of which would be calculated to diminish danger.

